# Dicarbonyls and glyoxalase in disease mechanisms and clinical therapeutics

**DOI:** 10.1007/s10719-016-9705-z

**Published:** 2016-07-12

**Authors:** Naila Rabbani, Mingzhan Xue, Paul J. Thornalley

**Affiliations:** 1 Warwick Systems Biology Centre, Coventry House, University of Warwick, Coventry, CV4 7AL UK; 2 Glyoxalase Research Group, Clinical Sciences Research Laboratories, Warwick Medical School, University of Warwick, University Hospital, Coventry, CV2 2DX UK

**Keywords:** Methylglyoxal, Glycation, Glyoxalase, Obesity, Diabetes, cancer, Renal failure, Cardiovascular disease, Therapeutics

## Abstract

The reactive dicarbonyl metabolite methylglyoxal (MG) is the precursor of the major quantitative advanced glycation endproducts (AGEs) in physiological systems - arginine-derived hydroimidazolones and deoxyguanosine-derived imidazopurinones. The glyoxalase system in the cytoplasm of cells provides the primary defence against dicarbonyl glycation by catalysing the metabolism of MG and related reactive dicarbonyls. Dicarbonyl stress is the abnormal accumulation of dicarbonyl metabolites leading to increased protein and DNA modification contributing to cell and tissue dysfunction in ageing and disease. It is produced endogenously by increased formation and/or decreased metabolism of dicarbonyl metabolites. Dicarbonyl stress contributes to ageing, disease and activity of cytotoxic chemotherapeutic agents. It contributes to ageing through age-related decline in glyoxalase 1 (Glo-1) activity. Glo-1 has a dual role in cancer as a tumour suppressor protein prior to tumour development and mediator of multi-drug resistance in cancer treatment, implicating dicarbonyl glycation of DNA in carcinogenesis and dicarbonyl-driven cytotoxicity in mechanism of action of anticancer drugs. Glo-1 is a driver of cardiovascular disease, likely through dicarbonyl stress-driven dyslipidemia and vascular cell dysfunction. Dicarbonyl stress is also a contributing mediator of obesity and vascular complications of diabetes. There are also emerging roles in neurological disorders. Glo-1 responds to dicarbonyl stress to enhance cytoprotection at the transcriptional level through stress-responsive increase of Glo-1 expression. Small molecule Glo-1 inducers are in clinical development for improved metabolic, vascular and renal health and Glo-1 inhibitors in preclinical development for multidrug resistant cancer chemotherapy.

## Dicarbonyl stress and the glyoxalase system

**Fig. 1:**
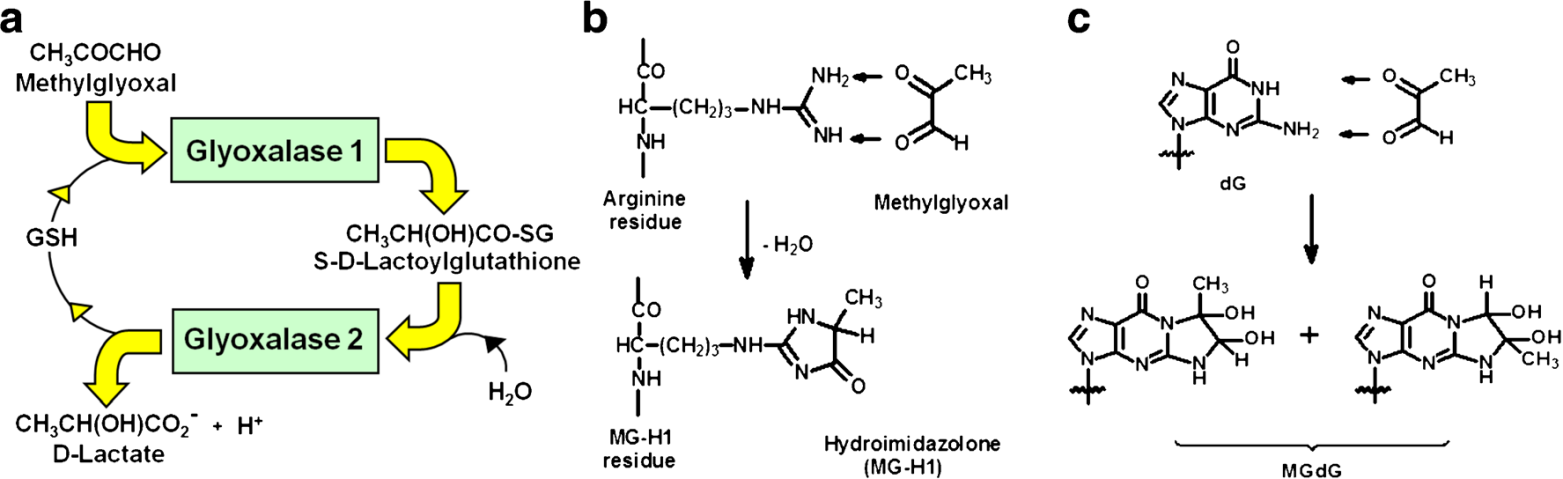
Biochemistry of dicarbonyl stress. **a** Metabolism of MG by the glyoxalase system. **b** Formation of hydroimidazolone MG-H1 from arginine residues. **c** Formation of imidazopurinone MGdG in DNA. Adduct residue is shown with guanyl base only

Dicarbonyl stress is the abnormal accumulation of dicarbonyl metabolites leading to increased modification of protein and DNA contributing to cell and tissue dysfunction in ageing and disease [1]. Highly reactive dicarbonyl metabolites often mediating dicarbonyl stress in physiological systems are methylglyoxal (MG), glyoxal, 3-deoxyglucosone (3-DG) and others. The glyoxalase system is a cytoplasm enzymatic pathway, which metabolises the most highly reactive acyclic dicarbonyls – mainly MG and glyoxal. It thereby plays a major role in suppressing dicarbonyl stress in physiological systems, keeping dicarbonyl metabolites at low, tolerable levels. Typical concentrations of glyoxal and MG are 50–150 nM in human plasma and 1–4 μM in plant and mammalian cells [2–4]. When dicarbonyl concentrations increase beyond this there is potential for protein and cell dysfunction leading to impaired health and disease. Examples of dicarbonyl stress are the increased MG in ageing plants [2], increased MG-protein modification in ageing human lens [5], increased plasma and tissue concentration of MG in diabetes [6], and increased concentrations of MG and glyoxal in renal failure [4]. Dicarbonyl stress is caused by an imbalance of the formation and metabolism of dicarbonyl metabolites and also by increased exposure to exogenous dicarbonyls – Fig. 1a. In this review there is a particular but not exclusive focus on MG as it is a major contributor to dicarbonyl stress in physiological systems. Other recent reviews focussing mostly on dicarbonyl stress in obesity and diabetes have been given elsewhere [7, 8].

## Formation of methylglyoxal

**Fig. 2:**
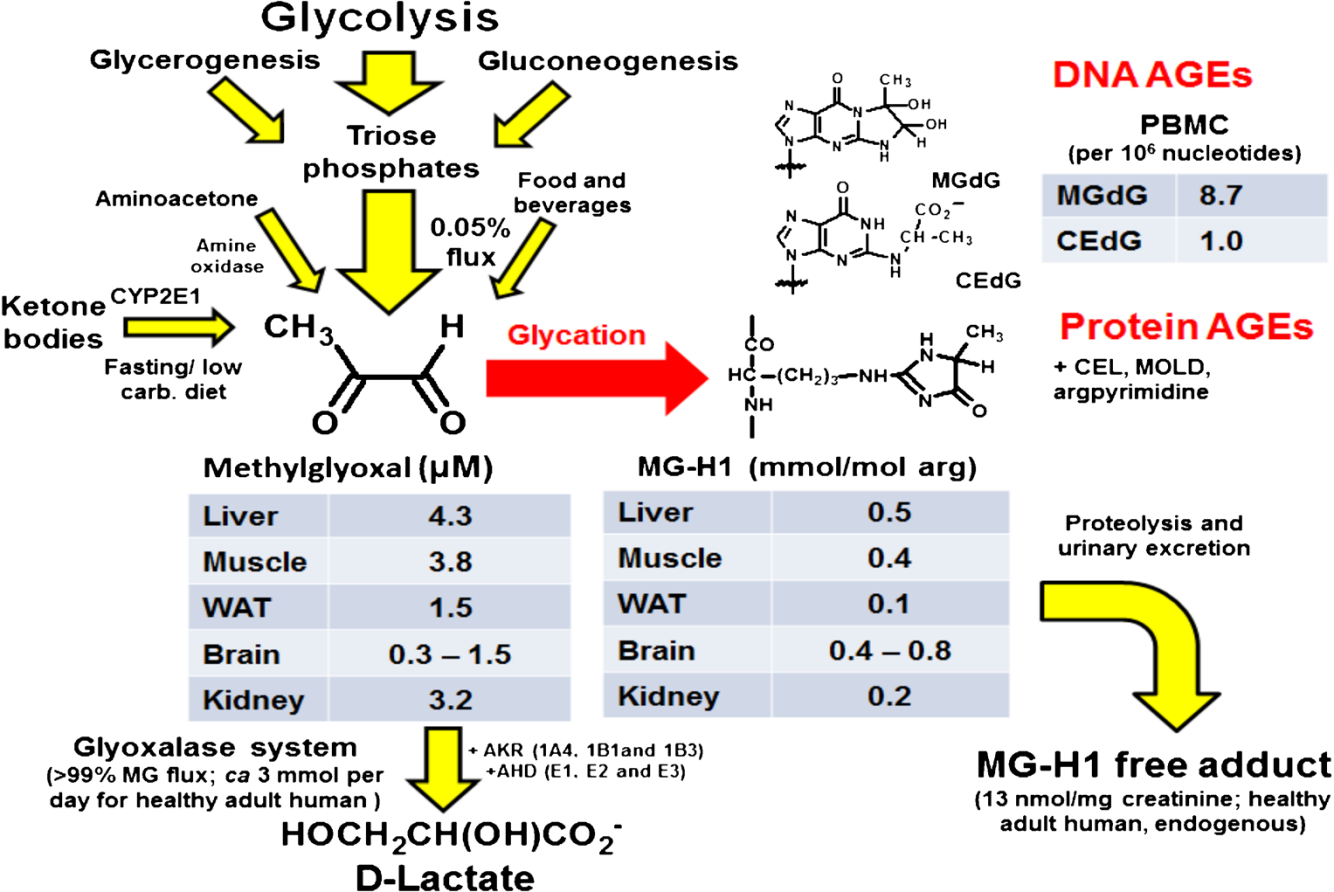
Formation of methylglyoxal, metabolism and glycation of protein and DNA *in vivo*. Tissue levels of MG and MG-H1 adduct residues in tissues are given for mice. PBMC DNA AGEs are for human subjects and flux of MG metabolised by the glyoxalase system and urinary excretion of MG-H1 free adduct is for healthy adduct humans. Data from [2, 63, 89, 122, 137] and Masania, J, Shafie, A, Rabbani, N and Thornalley, P.J., Unpublished observations

In mammalian metabolism, MG is formed at relatively high flux mainly by the trace level degradation of triosephosphates, glyceraldehyde-3-phosphate (GA3P) and dihydroxyacetonephosphate (DHAP) - 0.05 – 0.1 % of flux. GA3P was *ca.* 8-fold more reactive than DHAP in degrading to MG but as the concentration ratio of DHAP/GA3P in cells *in situ* is *ca.* 9 or similar [9], both of these triosephosphates are important sources of MG formation in physiological systems *in situ* [10]. MG formation is a minor fate of triosephosphates: early studies with red blood cells suggested only 0.089 % glucotriose (2 x glucose consumption) was converted to MG [11] and our subsequent studies with endothelial cells and fibroblasts suggest a similar flux. The rate of total cellular formation of MG was estimated to be *ca.* 125 μmol/kg cell mass per day [11], which for an adult human of 70 kg body mass and 25 kg body cell mass [12] equates to a predicted whole body rate of formation of *ca.* 3 mmol MG per day (or *ca.* 3 mg/kg body weight/day). MG is also formed by the oxidation of acetone catalysed by cytochrome P450 2E1 in the catabolism of ketone bodies [13] – which is low except where ketone bodies are increased as in diabetic ketoacidosis, prolonged (>3 days) fasting or low calorie diet [13–15]. MG may also be formed from the oxidation of aminoacetone by semicarbazide amine oxidase (SSAO) in the catabolism of threonine [16]. Recent estimates of the concentration and rates of metabolism of aminoacetone in the presence and absence of SSAO inhibitor suggest this pathway has a flux of *ca.* 0.1 mmol MG per day in human subjects [17] or *ca.* 3 % of total MG formation. Vascular adhesion protein-1 is considered the origin of SSAO activity in mammals *in vivo* [18]. It is found in plasma, endothelium, adipose tissue and smooth muscle and increases *ca.* 2-fold in congestive heart failure, diabetes and inflammatory liver diseases [19], and may relatedly increase MG formation in these conditions. MG is also formed by the degradation of proteins glycated by glucose and the degradation of monosaccharides [20]. Under physiological conditions with low level phosphate and chelation of trace metal ion, the predicted flux of MG formation from glycated protein degradation is *ca.* 0.2 mmol MG per day or *ca.* 7 % of total MG formation. Dietary contributions to MG exposure from food are normally relatively low: sweetened soft drink, 330 ml – 0.1 μmol MG [21], fruit juice, 330 ml – 0.7 μmol, bread/cakes, 100 g, 1–2 μmol and other foodstuffs [22]; that is, combined likely <0.03 mmol MG per day or <1 % MG exposure. MG in foodstuffs was also metabolised and/or reacted with proteins before absorption in the gastrointestinal tract and imposed dicarbonyl stress mainly in the gastrointestinal lumen [23]. The diet may contribute markedly greater to total exposure for other dicarbonyls where culinary heating is a source of formation; for example, 3-DG [24]. Sources of formation of MG and routes of metabolism are summarised in Fig. 2.

## Dicarbonyl metabolism by the glyoxalase system

Glyoxal and MG are metabolised mainly by glyoxalase 1 (Glo-1) of the glutathione-dependent glyoxalase system, with minor metabolism by aldoketo reductases (AKRs) and aldehyde dehydrogenases (ADHs). As total MG-derived glycation adducts excreted in urine of healthy human subjects was typically *ca.* 10 μmol per day [25, 26], it can be inferred that less than 1 % MG formed endogenously modifies the proteins. Most of the MG formed (>99 %) is metabolised by glyoxalase 1 (Glo-1) and aldoketo reductase (AKR) isozymes, which thereby constitute an enzymatic defence against MG glycation. From studies of the level of expression of Glo-1 and AKRs [27, 28], it can be inferred that Glo-1 activity *in situ* exceeds that of AKR activity for MG metabolism by >30-fold in all human tissues except the renal medulla where the expression of AKR is extraordinarily high. Indeed, Glo-1 is a highly efficient and high abundance enzyme; typically 0.02 % of total protein [27] and is in the top 13 % of proteins by abundance in human cells [29]. In principle, ADHs may also metabolise glyoxal and MG to glyoxylate and pyruvate, respectively. In examination of ADH-linked MG dehydrogenase activity in human cells to date we have found very low or undetectable activity. AKRs and ADH catalyse the metabolism of 3-DG whereas Glo-1 does not [4, 30]. Other proteins, “glyoxalase III” and DJ1, were proposed as glyoxalases but their low catalytic efficiency and cellular content suggests this is unlikely [31].

Basal and inducible expression of Glo-1, AKRs and ADH are under stress-responsive control by nuclear factor erythroid 2–related factor 2 (Nrf2) through regulatory antioxidant response elements (AREs) [32–36]. The cytoprotective function of Nrf2 therefore involves enhancing basal and inducible expression and activities of enzymes of dicarbonyl metabolism and thereby prevention of dicarbonyl stress [32]. Other regulatory elements in the mammalian GLO-1 gene are: metal response element, insulin response element, early gene 2 factor-isoform-4, and activating enhancer binding protein-2α, as reviewed [31]. Glo-1 expression is negatively regulated by hypoxia-inducible factor-1α (HIF1α) in hypoxia [37] and also by the receptor for advanced glycation endproducts (RAGE) [31]. Hypoxia may be an important physiological driver of dicarbonyl stress as it both increases MG formation by flux through anaerobic glycolysis and likely decreases Glo-1 expression through activation of HIF1α.

Glo-1 expression is also increased by copy number variation (CNV) of the GLO-1 gene in human and mouse genomes. Human GLO-1 is located in chromosome 6 at locus 6p21.2 [38] and mouse *Glo-1* in chromosome 17 at locus 17 a3.3 [39]. Gene cloning and bioinformatics analysis showed that human GLO-1 coding regions consists of 12 kb with introns separating six exons [40, 41]. CNV of human GLO-1 was detected by Redon *et al.* with a prevalence of *ca.* 2 %. [42]. Murine *Glo-1* CNV was found in inbred strains of mice which included complete copies of Glo-1 and complete and partial copies of other genes [43, 44], giving rise to a 2–4-fold increase in Glo-1 expression. GLO-1 duplication appeared linked to anxiety-like behaviour in mice but may rather be due to a proximate genetic locus co-duplicated with Glo-1 [45].

At *in situ* concentrations of MG, GSH and Glo-1, the formation and fragmentation of the hemithioacetal of MG and GSH are rapid compared to the Glo-1-catalysed step [31]. A consequence of this is that *in situ* activity of Glo-1 is directly proportional to GSH concentration. So cellular GSH concentration is an influential factor on *in situ* activity of Glo-1 and oxidative or non-oxidative depletion of GSH leads to increased glyoxal and MG [46]. The concentration of *S*-D-lactoylglutathione (SLG) is also maintained at low levels. This is likely so that lactoyl-transfer to protein thiol groups and related inactivation of enzymes with functional cysteinyl thiols is prevented [31]. SLG is also toxic if it leaks out of cells and is metabolised by γ-glutamyl transferase and dipeptidase with rearrangement to *N*-lactoylcysteine which is an inhibitor of pyrimidine synthesis [47]. A mathematical model of the glyoxalase pathway predicted very low levels of MG and SLG in cells which fitted well with experimental observation [31].

The likely evolutionary pressure for development of Glo-1 was to evolve an enzyme that accepts the major solution species of MG, the MG-GSH hemithioacetal, which is also highly efficient with k_cat_/K_M_ at the diffusion limit. Glo-1 is thereby exquisitely suited to its function. High reactivity of arginine and cysteine residues in Glo-1 protein could be a problem for Glo-1 stability which would occur with relatively low microscopic pKa. Our computations of microscopic pKa values (as previously described [48]) indicates functionally important R37, R122 and C139 of human Glo-1 have high microscopic pKa values (>12), which confers low reactivity towards MG. Also in cells with 1–4 μM MG, there is a pool of *ca.* 20 mM cysteinyl thiol groups and *ca.* 80 mM arginine residues to which MG may bind and only 2 μM and 4 μM of these, respectively, are functionally important residues of Glo-1 [27]. Therefore, Glo-1 is very resistant to inactivation *in situ* by MG.

## Biochemical consequences of dicarbonyl stress

Dicarbonyl stress produces increased *in situ* rates of glycation by dicarbonyls of proteins, DNA and basic phospholipids. Reaction with proteins is directed to arginine residues forming dihydroxyimidazolidine and hydroimidazolone adducts. The hydroimidazolone derived from MG, MG-H1, is one of the most quantitatively and functionally important AGEs in physiological systems – Fig. 1b. There are also minor lysine-derived AGEs formed: N_ε_-carboxymethyl-lysine and N_ε_(1-carboxyethyl)lysine formed from glyoxal and MG, respectively, and others. The major source of CML formation, however, is the oxidative degradation of N_ε_-fructosyl-lysine residues [49].

Dicarbonyl glycation is particularly insidious as it is directed to arginine – the amino acid residue with highest probability of location in functional sites of proteins, modification induces loss of charge of the side chain guanidino group and functionally important arginine residues tend to be those most reactive towards dicarbonyl glycation [50]. The extent of glycation of proteins by dicarbonyls is low, usually 1–5 %, but may increase in ageing and disease. Proteins modified by glyoxal and MG in dicarbonyl stress are recognised as mis-folded and directed to the proteasome for proteolysis. In yeast an unfocussed gene deletion analysis showed strains deleted for genes of ubiquitin-dependent protein degradation were sensitive to glyoxal and MG toxicity [51]. Examples of physiological dysfunction mediated by dicarbonyl glycation of arginine residues of proteins are: mitochondrial protein dysfunction and increased formation of reactive oxygen species (ROS) [52], inflammatory protein expression (RAGE, S100 proteins and high mobility group box-1) [53], mitochondrial pathway activated apoptosis [54] and cell detachment from the extracellular matrix and anoikis [55].

A surprising observation was increased chaperone function of αA-crystallin with very high modification by MG for reversing dithiothreitol- and heat-induced misfolding of proteins [56]. With lower, physiological extent of modification by MG, however, the chaperone function of αA-crystallin was not enhanced further [57].

Early studies of specific proteins modified by MG used antibodies to the trace MG-derived AGE, argpyrimidine. In endothelial cells the heat shock protein-27 (HSP27) was detected as a major MG modified protein [58]. This could not be verified by ultrahigh resolution Orbitrap mass spectrometry with direct examination for MG-modification in tryptic peptides – even when modification of proteins was increased 10-fold in cell lysates [59], suggesting earlier studies suffered interference. Subsequent studies showed recombinant HSP27 was modified by 500 μM – 5 mM MG at multiple sites and MG-modified HSP27 was more protective than unmodified protein against apoptotic cell death [60]. The physiological significance is unclear, however, as recent studies were unable to find evidence of MG modification of HSP27 [59] and increased MG and Glo-1 inhibitors tend to promote rather than suppress apoptosis [61, 62].

Glyoxal and MG are important precursors of DNA adducts in physiological systems: major adducts are imidazopurinones GdG and MGdG – nucleotide AGEs. MGdG was the major nucleotide AGE found physiologically – Fig. 1c. DNA content of MGdG exceeded those of the major DNA oxidative damage adduct, 8-hydroxydeoxyguanosine. Increased nucleotide AGEs was associated with DNA strand breaks and mutagenesis [63].

## Physiological consequences of dicarbonyl stress

Where dicarbonyl stress occurs there is potential for increased cell dysfunction, detachment from the extracellular matrix and anoikis, and apoptosis. Cell dysfunction is driven by the effects of protein glycation: loss of substrate protein activity – through inactivation and/or increased rate of proteolysis and decreased half-life (unless compensatory increased expression is activated), or by gaining a new and damaging function – for example, low density lipoproteins become small, dense and atherogenic by MG modification [64]. This affects multiple proteins – the dicarbonyl proteome - in different cell and tissue compartments [50]. The effects span multiple compartments by diffusion of increased MG or other dicarbonyl in dicarbonyl stress. MG permeates cell plasma membranes by passive diffusion of the unhydrated form. This is rate limited by MG dehydration, giving a half-life of ~4 min [2]. The half-life of MG for metabolism by the glyoxalase system to D-lactate from *in situ* rates of D-lactate formation in cells is *ca.* 10 min with free MG mostly (>95 %) reversibly bound to protein. The rate of irreversible binding to protein in plasma was *ca.* 3.6 h [65]. This implies that part of the MG formed inside cells leaks out from the site of formation and may diffuse through interstitial fluid into plasma and thereafter permeate back into interstitial fluid and cells of other tissues. Also, MG formed from the degradation of glycated proteins in the extracellular compartment may enter cells for metabolism by Glo-1 and AKRs. The locus of dicarbonyl stress and related pathogenesis linked to MG accumulation is therefore likely particularly sensitive to local decrease of Glo-1 expression and activity. Kinetic considerations of the rate of glycation of protein, similar to those for ROS [66], indicates a diffusion distance of MG of *ca.* 2–3 cm before irreversible reaction, suggesting that MG has relatively long range and half-life to locate and modify sensitive sites of proteins, often leading to protein inactivation and dysfunction.

## Dicarbonyl stress in ageing and disease

### Ageing

The link of dicarbonyl stress to ageing was unequivocally established in a functional genomics study of Glo-1 in the nematode *C. elegans* [52]. Ageing-related decline in renal function was prevented in transgenic rats overexpressing Glo-1 [67]. Intuitively we expect this is due to AGE accumulation in proteins of tissues and body fluids with related protein dysfunction. MG-derived AGEs were increased in human lens with age and this was linked to cataract formation [5, 68] and also in skin but to markedly lower extent [68]. Decreased Glo-1 activity was associated with age-linked impairment of wound healing [69] and increased dicarbonyl stress is associated with several ageing-linked diseases – see below. Glo-1 activity declines with age so there is also increased stress on cell proteolysis and compensatory gene expression to keep AGE-modified proteins to a low tolerable level and provide replacement unmodified proteins. Dicarbonyl stress is likely a feature of proliferative senescence of fibroblasts in culture where Glo-1 expression is decreased and glycolytic flux is increased [70, 71]. It is also likely involved in senescence of plants: dicarbonyl content of broccoli increased with age [2] and MG-H1 was a major AGE in *Arabidopsis* leaves [72].

### Obesity

For many years a genetic linkage of *Glo-1* to body weight in mice [73] and of GLO-1 to upper-arm circumference and supra-iliac skinfold thickness in human subjects [74] suggested a role for Glo-1 in obesity. In the mouse overeating model of obesity, leptin mutant (ob/ob) mice, Glo-1 protein was decreased 80 % in the liver [75]. Recent conference reports described increased weight gain on high fat diet (HFD)-fed mouse with through-life expression of GLO-1 siRNA and Glo-1 deficiency, compared to wild-type controls [76], and decreased weight gain in Glo-1 overexpressing transgenic mice [77], suggesting a functional role of Glo-1 and dicarbonyl stress in obesity. HFD-fed wild-type mice had increased MG-H1 content of heart and liver, as judged by immunoassay [78]. Dicarbonyl stress may be a mediator of obesity and insulin resistance and thereby a risk factor for development of type 2 diabetes mellitus (T2DM) and non-alcoholic fatty liver disease (NAFLD).

### Diabetes and diabetic vascular complications

Hypotheses for the involvement of dicarbonyl stress in disease are most advanced and critically evaluated for involvement in the vascular complications of diabetes. Glo-1 activity is decreased and MG-H1 residue content of proteins is increased in the kidney, retina and nerve of pre-clinical models of microvascular complications of diabetes (nephropathy, retinopathy and neuropathy) [79–83]. Functional genomics studies with Glo-1 deficient mice and Glo-1 overexpressing transgenic mice and preventive intervention such as high dose thiamine and Benfotiamine support increased MG as a risk factor linked to the development of diabetic microvascular complications [79, 84–87]. Dicarbonyl stress is also linked to diabetic cardiovascular disease – see below. Formation of MG is increased in cells with GLUT1 glucose transport incubated in high glucose concentration [11, 88]. Decreased Glo-1 activity synergises with increased MG formation to increase cellular and extracellular MG concentration. MG content of blood samples was increased by up to 5–6 fold in patients with diabetes [6]. Increased MG has been found in diabetic kidney *in vivo* [89] and vascular endothelial cells in high glucose concentration cultures *in vitro* [55] – including in mitochondria [90]. In locations where it is difficult to excise tissues without leaking of MG from cells and disrupting extracellular fluid – such as retina and cellular and extracellular compartment of peripheral nerve, evidence of dicarbonyl stress is increased levels of dicarbonyl–derived AGEs [83, 91]. Increased MG concentration and protein content of MG-H1 was not found in liver, skeletal muscle and brain in experimental models of type 1 diabetes [25, 83, 92]. MG-derived AGEs were increased in plasma protein and skin collagen of diabetic patients and were linked to risk of microvascular and macrovascular complications [26, 93–95].

Dicarbonyl stress may also play a role in development of T2DM through promotion of insulin resistance [77, 96] and development of type 1 diabetes through mediation of beta-cell toxicity [97]. Recent studies have found increased levels of MG, glyoxal and 3-DG of subjects with impaired glucose tolerance and patients with T2DM in the fasting state and during an oral glucose tolerance test challenge [98]; plasma and glyoxal levels were *ca.* 3-fold higher for MG and glyoxal than obtained using the reference assay protocol which control [2].

### Chronic renal disease

Experimental models of renal failure, bilateral nephrectomy and bilateral ureteral ligation - models of acute total loss and partial loss of renal function, respectively, were associated with profound dicarbonyl stress. Plasma glyoxal and MG increased 5 and 15 fold within 72 h [99]. Patients with end stage renal disease (ESRD) on hemodialysis and peritoneal dialysis had increased plasma MG and flux of formation of dicarbonyl-derived AGEs [4, 100]. The cause of dicarbonyl stress is unlikely due to decreased dicarbonyl excretion as there is little in normal health [23], although it is linked to renal function [99]. Decreased Glo-1 expression by hypoxia and inflammation in ESRD [101, 102], hypoxia-induced increased anaerobic glycolysis [103] and decreased disposal of triosephosphates by the reductive pentosephosphate pathway (enzymes of which are inhibited by uremic toxins [104]) leading to increased formation of MG may produce dicarbonyl stress in ESRD. Decreased Glo-1 activity in rare GLO-1 frameshift mutation heterozygote human subjects was associated with decreased glomerular filtration rate [67]. A patient with ESRD and low Glo-1 activity had a high frequency of recurrent cardiovascular disease (CVD) events [105]. Further studies showed a high mortality rate in patients with homozygous GLO-1 419CC mutation - reviewed in [106]. This suggests a link of dicarbonyl stress to development of renal failure and CVD complications of ESRD.

### Cardiovascular disease

A recent pre-clinical and clinical integrative genomics study revealed Glo-1 deficiency as a driver of CVD [107]. Chemical inhibition of Glo-1 induced atherosclerosis in apoE deficient mice [108]. MG-derived AGEs in plasma protein have been found to be linked to risk of CVD in diabetes [94, 109]. High levels of MG-H1 and CML were associated with rupture-prone plaques in human carotid endarterectomy, accumulating in macrophages surrounding the necrotic core. The expression of Glo-1 was decreased in ruptured compared with stable plaque segments [110]. However, overexpression of Glo-1 in mice did not affect atherosclerotic lesion size and severity in ApoE−/− mice with or without diabetes [111]. Unexpectedly plasma MG and glyoxal concentration were not decreased in Glo-1 transgenic mice. The reason for this is not clear but dicarbonyls are predominately sourced from metabolism and there was limited increase in Glo-1 activity of the liver in the Glo-1 transgenic mice [112]. Dicarbonyl stress in plasma likely contributes to CVD risk through induction of dyslipidaemia and vascular cell dysfunction. MG modification of LDL induced atherogenic transformation to small, dense LDL with increased affinity for arterial walls through binding to heparan sulfate proteoglycans [64]. MG modification of HDL induced re-structuring of the HDL particles, increasing density, decreasing stability and plasma half-life *in vivo* [113]. Dicarbonyl stress also induces vascular cell dysfunction: silencing of Glo-1 in human aortal endothelial cells changed expression of >400 genes – including increased expression of RAGE and associated ligands [53, 107].

### Carcinogenesis, tumour growth and cancer chemotherapy

Dicarbonyl stress has a duality of function in cancer development and treatment. Glo-1 is a tumour suppressor protein and a mediator of multidrug resistance (MDR) in cancer chemotherapy. The tumour suppressor function of Glo-1 was revealed in a genome-wide study in p53 knockout, Ras overexpression model of liver carcinogenesis [114]. This is consistent with mutation arising from of dicarbonyl glycation of DNA sometimes leading to carcinogenesis. There were 14 tumour suppressor genes found and currently *Glo-1* is the only one for which a readily available strategy for increased cancer prevention exists – dietary Glo-1 inducers in functional foods. The role of Glo-1 in MDR was revealed in a transcriptome-wide subtraction technique of drug-sensitive and drug-resistant tumour cell lines [115]. Increased Glo-1 expression in tumours may be mediated through GLO-1 amplification [116], and also by mutation and increased transcriptional activity of Nrf2 through ARE-linked up-regulation of Glo-1 transcription [117]. High Glo-1 activity may be permissive for growth with high glycolytic activity and flux of MG formation [63]. MDR tumours are susceptible to cell permeable Glo-1 inhibitors [116, 118].

### Other diseases

Following discovery of Glo-1 gene duplication in some strains of mice with an anxiety phenotype [43], a link of Glo-1 to pathologic anxiety was proposed – although the anxiety phenotype was linked to both increased and decreased Glo-1 expression [119, 120]. In attempts to link Glo-1 metabolically to dysfunctional brain metabolism, MG was found to agonise the GABA_A_ receptor in primary cerebellar granule neurons with a median effective concentration EC_50_ of 10.5 μM and this was proposed as mediator of sedation to explain increased GLO-1 CNV with an anxiety phenotype [121]. MG concentration in mouse brain tissue is *ca.* 7-fold lower than this [122] and only approached the EC_50_ value with dosing of 300 mg/kg MG [121] – similar to doses producing acute toxicity [123]. Transgenic mice with 2-fold, 4-fold and 5-fold increased Glo-1 expression had an anxiety phenotype with 4- and 5-fold increased expression but not with 2-fold increased expression [121]. Some inconsistences remain, therefore, and further investigation is required.

Dicarbonyl stress has also been linked to severe schizophrenia through a rare frameshift mutation of GLO-1 [124], synucleinopathies such as Parkinson’s disease in experimental pre-clinical models [122] and Alzheimer’s disease through clinical biomarker studies [125].

### Dicarbonyl stress-based therapeutics

#### Alleviation of dicarbonyl stress by glyoxalase 1 inducers

Initial attempts to alleviate dicarbonyl stress were made through dicarbonyl scavengers and claimed for dicarbonyl scavenging properties of existing drugs in treatments for vascular complications of diabetes. A challenge in the design of dicarbonyl scavengers is to achieve sufficient reactivity for the low concentration of drug achieved clinically with the 1000–10,000 fold higher concentration of arginine residues in tissues and body fluids. Aminoguanidine and phenacylthiazolium bromide showed some promise but their toxicity and instability prohibited further development [4, 126, 127]. Metformin and pyridoxamine are not efficient scavengers of MG and where associated with alleviation of dicarbonyl stress likely function by other mechanisms [128–131]. High dose thiamine supplements for prevention of T2DM and vascular complications of diabetes may work partly by preventing the formation of MG through increased disposal of triosephosphates in the reductive pentosephosphate pathway [132, 133].

A better strategy is development of Glo-1 inducers through activation and binding of Nrf2 to the GLO-1 functional ARE [32]. This offers an alternative that is likely safe and effective. It also addresses a key cause of dicarbonyl stress in disease – disease-associated, tissue-specific deficiency of Glo-1. Detection of Nrf2 activators for ARE-linked induction of Glo-1 expression requires a specific screen with the GLO-1-ARE motif or similar as not all Nrf2 activators induce expression of Glo-1. Nrf2 activators typically change expression of a subset of ARE-linked genes. The basis of this subset selection is unknown but it is likely determined by the level of functionally-active Nrf2 achieved in the cell nucleus in response to the Nrf2 activator, recruitment of relevant accessory proteins – such as small maf proteins [134, 135], and absence of activation of counter-signalling effects required to induce the ARE-linked gene of interest. Nrf2 regulates inducible expression of *ca.* 890 genes [136]. The advantage of Nrf2 regulated, ARE-regulated genes is that they are a battery of protective genes and so where co-regulated along with Glo-1, they tend to add to the health beneficial response. In this regard, concurrent induction of GSH synthesis for increased cellular GSH concentration to support increased *in situ* activity of Glo-1 is particularly beneficial [137].

A Glo-1 inducer formulation has been optimised and evaluated in Phase 1 clinical trial (Clinicaltrials.org; NCT02095873) in overweight and obese subjects for safety and target pharmacology at a working dose. We also did functional assessments – Phase 2 A trials for health improvement in obesity. The Glo-1 inducer is a binary combination of *trans*-resveratrol and hesperetin (tRES-HESP) and was evaluated in a randomised, placebo-controlled crossover clinical trial in 29 overweight and obese subjects. In highly overweight subjects (BMI >27.5 kg/m^2^), tRES-HESP co-formulation increased expression and activity of Glo-1, decreased plasma methylglyoxal and total body methylglyoxal-protein glycation. It decreased fasting and postprandial plasma glucose, increased oral-glucose-insulin-sensitivity (OGIS) index – an assessment of insulin sensitivity, and improved arterial dilatation. In all subjects, it decreased vascular inflammation marker sICAM-1. In previous clinical evaluations, tRES and HESP individually were ineffective. tRES-HESP co-formulation could be a suitable treatment for improved metabolic and vascular health in overweight and obese populations. It now available for evaluation in Phase 2 clinical trial against disease targets.

This first-in-class Glo-1 inducer trial establishes Glo-1 target pharmacology for tRES-HESP. Whilst increased Glo-1 expression likely contributes to the observed health beneficial effects, changes in other gene expression occurred and their interplay may also mediate the overall health benefit achieved. Nevertheless it was pursuit and optimisation of induction of Glo-1 expression that arrived at this synergistic combination of bioactive compounds and achieved improved metabolic and vascular health in overweight and obese subjects that is unmatched by other therapy. The marked health improvements were achieved with expression of many antioxidant-linked genes unchanged – at least in the peripheral blood mononuclear cells analysed.

Cell permeable Glo-1 inhibitors which increase dicarbonyl stress may find use as anti-tumour and anti-microbial agents for treatment of Glo-1-linked MDR tumours and microbial infections. *S-p*-Bromobenzylglutathione diethyl ester was the first cell permeable Glo-1 inhibitor developed and had median growth inhibitory concentration GC_50_ values in the range 7–20 μM for a range of cancer cell lines. Subsequent development of the cyclopentyl ester derivative increased potency and had anti-tumour activity in tumour-bearing mice [62, 138, 139]. A difficulty in clinical translation is identifying tumours that are sensitive to Glo-1 inhibitors. These are likely tumours with a relatively high flux of MG formation and high activity of Glo-1 such that when a Glo-1 inhibitor is delivered into the tumour, MG accumulates rapidly to toxic levels. GLO-1 amplification in tumours is not a reliable marker of this as it is not always functional and does not report on flux of MG. Systems modelling of the glyoxalase pathway is beneficial in assessment of the potency of Glo-1 inducer or Glo-1 inhibitor required to achieve the desired pharmacological and therapeutic effects [31].

Stages of development of therapeutics targeting dicarbonyl stress are summarised in Table 1.

**Table 1:** Therapeutic agents in development targeting the glyoxalase system

Therapeutic class	Mechanism of action	Primary target application (secondary)	Stage of development	Reference
Glyoxalase 1 inducer	Small molecule Nrf2 activator targeting GLO-1-ARE transcriptional activity	Diabetic nephropathy (other microvascular complications of diabetes; obesity – NAFLD; cardiovascular disease)	Clinical trial Phase 2 ready (Phase 1 complete with safety, dose and pharmacology established).	[137]
Glyoxalase 1 inhibitor	Substrate analogue inhibitor diester (prodrug)	Cancer (GLO-1 overexpressing, MDR tumours)	Pre-clinical *in vivo* models	[62, 118, 140]

## Abbreviations

ADH: Aldehyde dehydrogenase
AGE: Advanced glycation endproduct
AKR: Aldoketo reductase
ARE: Antioxidant response element
CVD: Cardiovascular disease
3-DG: 3-Deoxyglucosone
DHAP: Dihydroxyacetonephosphate
ESRD: End stage renal disease
GA3P: Glyceraldehyde-3-phosphate
GdG: 3-(2′-deoxyribosyl)-6,7-dihydro-6,7-dihydroxyimidazo-[2,3-b]purin-9(8)one
Glo-1: Glyoxalase 1
HFD: High fat diet
HIF1α: Hypoxia-inducible factor-1α
HSP27: Heat shock protein 27
MDR: Multidrug resistance
MG: Methylglyoxal
MGdG: 3-(2′-deoxyribosyl)-6,7-dihydro-6,7-dihydroxy-6/7-methylimidazo-[2,3-b]purine-9(8)one
MG-H1: N_δ_-(5-hydro-5-methyl-4-imidazolon-2-yl)-ornithine
NAFLD: Non-alcoholic fatty liver disease
Nrf2: Nuclear factor erythroid 2–related factor 2
PBMC: Peripheral blood mononuclear cells
RAGE: Receptor for advanced glycation endproducts
ROS: Reactive oxygen species
SSAO: Semicarbazide amine oxidase
T2DM: Type 2 diabetes mellitus
